# Association between Eating Speed and Classical Cardiovascular Risk Factors: A Cross-Sectional Study

**DOI:** 10.3390/nu11010083

**Published:** 2019-01-04

**Authors:** Indira Paz-Graniel, Nancy Babio, Ignacio Mendez, Jordi Salas-Salvadó

**Affiliations:** 1 Department of Biochemistry and Biotechnology, Human Nutrition Unit, Universitat Rovira i Virgili, Institut d’Investigació Sanitària Pere i Virgili (IISPV), Hospital Universitati Sant Joan de Reus, Reus 43201, Spain; nut.indirapaz@gmail.com (I.P.-G.); ignacioagustinmg@gmail.com (I.M.); jordi.salas@urv.cat (J.S.-S.); 2 Consorcio CIBER, M.P. (CIBEROBN), Fisiopatología de la Obesidad y la Nutrición, Instituto de Salud Carlos III (ISCIII), Madrid 28029, Spain

**Keywords:** eating speed, hypertriglyceridemia, PREDIMED study, metabolic syndrome

## Abstract

Cardiovascular disease (CVD) is one of the main causes of morbidity and mortality around the world. Lifestyle is recognized as a key factor in the development of metabolic disorders and CVD. Recently, eating speed has been of particular interest since some studies have associated it with the development of obesity and other cardiometabolic disorders. We aimed to assess the association between eating speed and various cardiovascular risk factors. We conducted a cross-sectional analysis within the framework of the PREDIMED (Prevención con Dieta Mediterránea) study with 792 participants from the Reus-Tarragona center. Eating speed was self-reported according to participant perception and categorized as slow, medium, or fast. The association between eating speed and cardiovascular risk factors was assessed using Cox regression models with constant time of follow-up for all individuals. Compared to participants in the slow eating speed category, those in the faster eating speed category were 59% more likely to have the hypertriglyceridemia component of the metabolic syndrome (MetS) (Hazard Ratio, (HR) 1.59; 95% Confidence Interval (CI) 1.16–2.17), even after adjustment for potential confounders (HR 1.47; 95% CI 1.08–2.02). No other significant differences were observed. Eating speed was positively associated with the prevalence of the hypertriglyceridemia component of the MetS in a senior population at high cardiovascular risk.

## 1. Introduction

Cardiovascular disease (CVD) is one of the main causes of morbidity and mortality in developed countries. Hypertension, dyslipidemia, type 2 diabetes mellitus, overweight/obesity, and metabolic syndrome (MetS) are considered risk factors for the development of CVD.

Various lifestyle factors such as physical activity, smoking, and diet have been recognized as important modifiable risk factors for CVD [1]. Therefore, several public health strategies promoting physical activity, smoking cessation, and a healthy dietary pattern have been developed for CVD prevention [2].

Recently, non-nutritional factors such as the number of daily meals, snacking, and eating speed or eating speed rate, have also been associated with an increased risk of obesity [3,4], abdominal obesity [5], and other cardiovascular risk factors [6].

In fact, a systematic review and meta-analysis of several cross-sectional [7,8,9,10] and longitudinal [11,12,13] studies, most of which were conducted in Asian populations, showed that eating quickly is associated with increased body mass index (BMI) and a greater risk of obesity [14]. However, the magnitudes of association across studies were significantly heterogeneous, something that was attributed to differences between the populations studied and to the methods used to measure eating speed (self-reported eating speed or self-reported eating rate) [14]. Furthermore, cross-sectional and longitudinal studies have revealed controversial findings on gender.

The mechanisms explaining these associations are unclear. High energy intake over a short period of time could interfere with satiety signals and consequently lead to weight gain and an increase in total energy consumption in the long term [15]. Through this mechanism, and perhaps others, eating speed might also have a negative effect on metabolism [16]. In fact, an association between eating speed and various cardiometabolic risk factors has also been reported in some studies [17].

Because of this heterogeneity between studies, the aim of our study was to cross-sectionally assess the associations between self-reported eating speed and the risk of obesity, dyslipidemia, MetS and its components in a senior Mediterranean population at high cardiovascular risk.

## 2. Materials and Methods

This cross-sectional study was carried out within the framework of the PREDIMED (Prevención con Dieta Mediterránea)-Reus study, a large, parallel-group, clinical trial aiming to assess the effects of the Mediterranean diet on the primary prevention of CVD, the design of which has been described in detail elsewhere [18,19,20].

Eligible participants were men and women aged 55 to 80 years with no previous history of cardiovascular disease and with either type 2 diabetes or at least three of the following CVD risk factors: current smoking, BMI ≥ 25 kg/m^2^; blood pressure ≥140/90 mmHg or treatment with antihypertensive medication; serum low density lipoprotein-cholesterol (LDL-c) ≥ 160 mg/dL or treatment with hypocholesterolemic agents; high density lipoprotein-cholesterol HDL-c ≤ 40 mg/dL in men and ≤50 mg/dL in women; or family history of early-onset CVD (≤55 years in men and ≤60 years in women).

The project was approved by the Ethical Committee of the Reus-Hospital de Sant Joan, and all the participants signed the corresponding informed consent. The PREDIMED trial was registered at http://www.controlled-trials.com (ISRCTN35739639). A detailed protocol has been published [20] and it is available online (http://www.predimed.es).

Participants were excluded if they did not complete the eating behavior questionnaire that was the object of this study, if they did not complete the baseline food frequency questionnaire (FFQ), or if they reported extreme total energy intakes with values outside the pre-specified limits (500–3500 kcal/day in women and 800–4000 kcal/day in men).

Participants completed the following: (a) a questionnaire on lifestyle variables, medical history, and medication use; (b) a 14-item validated questionnaire [21,22] designed to assess adherence to the MedDiet; (c) a validated 137-item semi-quantitative FFQ [23], (d) the validated Spanish version of the Minnesota Leisure-Time Physical Activity Questionnaire [24,25], and (e) a non-validated ad-hoc eating behavior questionnaire that included information about how they perceived their eating speed in the main meals classified in five categories (very slow, relatively slow, medium, relatively fast, very fast), the number of main meals they ate during the day, and whether they used dental prosthesis or not. The same information was collected from a sub-sample of 122 partners of the participants. The correlation coefficient between self-reported eating speed and the eating speed reported by his/her partner was 0.472, *n* = 122; *p* ≤ 0.001.

Anthropometrical variables and blood pressure measurements were determined by trained staff. Height and weight were measured with light clothing and no shoes. Waist circumference was measured midway between the lowest rib and the iliac crest. Blood pressure was measured using a validated oscillometer (Omron HEM705CP, Hoofddorp, The Netherlands) in triplicate with a 5-min interval between each measurement, and the mean of these values was recorded.

Blood samples were collected from all participants after a 12-hour overnight fast. Plasma glucose, serum HDL-c, and triglyceride levels were measured using standard enzymatic automated methods.

Overweight and obesity were defined using the WHO (World Health Organization) criteria: participants with a BMI between 25 and 29.9 kg/m^2^ were regarded as overweight, and those with a BMI ≥ 30 kg/m^2^ were regarded as obese [26]. MetS was defined in accordance with the updated harmonized criteria of the International Diabetes Federation and the American Heart Association/National Heart, Lung, and Blood Institute [27]. Individuals were diagnosed with MetS if they presented three or more of the following components: waist circumference >102 cm in men and >88 cm in women; serum triglyceride >150 mg/dL or drug treatment for elevated triglycerides; HDL-c <40 mg/dL in men and <50 mg/dL in women or drug use for low HDL-c; blood pressure ≥130/85 mmHg or antihypertensive drug treatment; and fasting plasma glucose level ≥100 mg/dL or hypoglycemic treatment.

The data are presented as means ± standard deviation (SD) for quantitative variables and as numbers and percentages for qualitative variables. The variables that did not present a normal pattern according to the Kolmogorov-Smirnov tests were transformed logarithmically prior to statistical analysis. We used Pearson’s chi squared test for qualitative variables and *t*-tests or one-way analysis of variance (if more than two variables were analyzed) for a quantitative independent variable and a quantitative dependent variable.

Given the cross-sectional design, Cox regression models with constant follow-up time (*t* = 1) for all individuals were fitted to estimate Hazard Ratio (HR) and 95% confidence intervals (CIs) and assess the association between eating speed and obesity, as well as MetS and/or its components. According to methodologists, this model is better suited than logistic regression to cross-sectional studies with frequent prevalent outcomes, such as the present study, since it avoids overestimating the prevalence ratios derived from the odds ratios when logistic regression is applied in analyses with frequent outcomes [28,29]. Because of the difference in the number of individuals in the various categories of eating speed, the slow and relatively slow categories, and fast and relatively fast categories were respectively merged, and the final categories used were slow, medium, and fast. For all models, the slow speed category was taken as a reference. Models were adjusted for the following possible confounding variables: age, sex (women or men), educational level (low-medium or high), smoking status (current, former, never smoker), use of dental prosthesis (yes or no), total energy intake (kcal/day), alcohol consumption (g/day), physical activity Metabolic Equivalent Task (MET/min/day), and adherence to MedDiet (categorized as low when participants scored <8 points or high when they scored 8≤ on the respective questionnaire).

The PREDIMED trial recently reported some slight departures from the randomization protocol that affected a small subset of participants [18]. However, a proper randomization was conducted at the PREDIMED-Reus recruitment site, in which only a small number of participants were directly allocated to the same arm of the trial as their relatives because a previous member of the same household (usually the spouse) had already been randomized in the trial (*n* = 29; 6.9% of the total sample). To address this issue, we applied robust estimates of the variance to correct for intra-cluster correlation in the Cox regression models.

All statistical tests were two-sided, and the significance level was set at *p* values ≤ 0.05. All analyses were performed using StataCorp. 2015. Stata Statistical Software: Release 14 (StataCorp LP, College Station, TX, USA).

## 3. Results

Of the 877 participants randomized in the Reus-Tarragona PREDIMED center, 73 were excluded because they had not completed the corresponding food behavior questionnaire. Twelve participants who reported extreme total energy intakes with values outside the pre-specified limits were also excluded. Therefore, the final sample was 792 participants (57% female and 43% male), with a mean age of 67.51 ± 5.86 years, and a mean BMI of 29.62 ± 3.32 kg/m^2^. In terms of eating speed, 22.9%, 31.7%, and 45.5% of the study participants were classified into slow, medium, and fast eating speed categories, respectively. The general and dietary characteristics of the participants in these categories are summarized in Table 1 and Table 2, respectively.

Participants who self-reported a fast eating speed were most frequently younger women who had higher values of diastolic blood pressure, BMI, and plasma triglycerides than those in the slow category. There were fewer participants with dental prosthesis in the fast-eater category than in the slow-eater category. In addition, individuals who self-reported a faster eating speed had a higher prevalence of hypertriglyceridemia than those in the slow category.

Individuals in the fast eating speed category consumed a significantly higher percentage of energy as protein and less total alcohol than those in the slower eating speed category. No differences in total energy and other nutrients intake were shown between categories of eating speed.

Table 3 shows the associations between eating speed categories and various cardiovascular risk factors. Compared to those in the slow category of eating speed, participants in the fast category showed an increased risk of obesity (HR 1.22; 95% CI 1.00–1.49). However, this association was not significant after adjusting for confounding factors (HR 1.15; 95% CI 0.94–1.40). In addition, individuals in the fast category had a significant 59% higher risk of hypertriglyceridemia (HR 1.59; 95% CI 1.16–2.17) than those in the slow category. This association remained statistically significant after confounding factors had been adjusted for in a multivariable model (HR 1.47; 95% CI 1.08–2.02). No statistically significant associations were observed with other cardiovascular risk factors.

In order to prove the robustness of the associations observed, we conducted sensitivity analysis by adjusting our models for the other four MetS individual components except the component of interest, and the association remained unchanged (data not shown). An additional text file shows this in more detail (see Supplementary Table S1).

## 4. Discussion

In this cross-sectional study, self-reported eating speed was positively associated with the prevalence of hypertriglyceridemia in a senior population at high cardiovascular risk, even after potential confounders had been adjusted for. However, no associations were observed between eating speed and the prevalence of obesity or MetS and its following components: central obesity, low HDL-c, high fasting glucose, and high blood pressure.

Investigating the association between eating speed and weight status, previous studies, mostly conducted in a middle-aged Asian population, have demonstrated a positive association between eating speed, BMI [7,8,9,10], and obesity prevalence [30,31]. This association has been reported in a healthy population but also in individuals with type 2 diabetes [32]. The only cross-sectional study to have been conducted in European men and women showed a positive association between self-reported eating rate and BMI. However, after adjusting for possible confounders, this association only remained in women [10]. Only two longitudinal studies in healthy populations—one from New Zealand [11] conducted in women, and the other from Japan [12] conducted in men—have analyzed the association between eating speed and weight change, and positive associations were reported in both. A recent longitudinal study conducted in a Japanese population with type 2 diabetes mellitus (T2DM) showed that those individuals eating slower at baseline had a lower prevalence of obesity [13] and had lower BMIs and waist circumferences than those eating fast after 6 years of follow-up. In contrast to this evidence, even though we observed significant differences in BMI and obesity prevalence among eating speed categories, the possible associations between these variables and eating speed categories did not reach statistical significance.

Only one case-control [33], one longitudinal [34], and two cross-sectional [9,17] studies have shown significant associations between eating speed and other cardiometabolic risk factors. In a study conducted on 30 non-alcoholic, non-diabetic, severely obese women, the eating rate positively correlated with the waist-hip circumference and levels of triglycerides [33]. In a cross-sectional analysis of Korean men, an inverse association between eating speed rate and HDL-c levels were observed [9]. In another cross-sectional study conducted on a Chinese population, investigators observed that eating speed might increase the risk of MetS, blood pressure, and central obesity for both genders. In addition, an increased risk of elevated triglycerides and reduced HDL-c levels for males and elevated fasting plasma glucose for females was observed [17]. In a three-year follow-up study [34], a higher incidence of MetS was observed in fast eaters than in slow eaters. Compared to the slow eating speed category, those participants in the highest category had a higher risk of abdominal obesity, hypertriglyceridemia, and low HDL-c level components of the MetS. However, no associations were reported for blood pressure and glucose.

The possible mechanisms linking the speed of eating and obesity, hypertriglyceridemia, and other metabolic alterations are unclear. As far as BMI and obesity status are concerned, it has been suggested that eating fast may contribute to a delayed feeling of fullness and satiety compared to eating slowly [8,35,36]. Accordingly, a randomized cross-over study examined the effect of eating speed at breakfast on appetite hormone response, total daily intake, and subjective appetite perceived by the participants. Desire to eat at 60 min was significantly lower in the slow than in the fast eaters, but no association was found between breakfast eating speed and peripheral appetite hormone concentrations (ghrelin, GLP-1, PYY), hunger, fullness, or daily energy intake [37]. Another cross-over clinical trial conducted on normal-weight and overweight participants compared the effect of consuming the same meal ad libitum, but at different speeds, and assessed the weight and energy content of the food as well as perceived hunger and fullness at different times using visual analog scales. In this study, eating slowly significantly lowered meal energy intake in the normal-weight group but not in the overweight/obese group, and led to lower hunger ratings in both groups and increased fullness ratings in the normal-weight group 60 min after the meal had begun [38]. Nevertheless, in our study we did not observe significant differences in total daily energy intake between self-reported eating speed categories. As far as hypertriglyceridemia is concerned, some authors suggested that the intake of a high amount of energy over a short period induces more sustained peaks in plasma glucose and insulin [16], which could favor an anabolic state that stimulates liver lipogenesis and therefore increases plasma triglyceride levels [33,39].

Our study has some limitations. First, our results came from a cross-sectional analysis and a causal relationship between eating speed and hypertriglyceridemia cannot be proven. Therefore, we cannot discount the possibility that the positive associations observed are due to other confounding factors not covered by the statistical analysis. Second, eating speed was self-reported, which may affect the validity of the results. Even so, when we studied the correlation between eating speed self-reported by the participants and the eating speed reported by their partners, a positive correlation was observed. Third, the present study focused on a senior population at high cardiovascular risk, therefore it is difficult to extrapolate our results to the general population. Finally, in our study we measured the self-reported eating speed (time perceived by the participants that they need to eat a main meal), but not the “eating speed rate” (determined by the total food consumption in gr/min or kcal/min) as other investigators have done.

## 5. Conclusions

Considering the aforementioned limitations, the results of our study suggest that eating faster increases the risk of hypertriglyceridemia, a recognized risk factor for CVD. Interventional and long-term studies are needed to clarify the cause–effect relationship between the speed of eating and BMI, or other cardiovascular risk factors. If these associations are proven, interventions aiming to decrease the speed of eating could be useful for designing strategies to fight cardiometabolic diseases.

## Figures and Tables

**Table 1:** General characteristics of the study population across self-reported eating speed categories.

	Eating Speed Categories			
	Slow *n* = 181	Medium *n* = 251	Fast *n* = 360	*p*^a^ Value
Women, % (*n*)	54.70 (99)	49.40 (124)	63.61 (229)	<0.01
Age, years	68.82 ± 5.72	68.20 ± 5.81	66.37 ± 5.78	<0.01
BMI (kg/m^2^)	29.19 ± 3.50	29.40 ± 3.14	30.00 ± 3.32	0.01
Obesity, % (*n*)	41.44 (75)	41.04 (103)	50.56 (182)	0.03
Waist circumference (cm)				
Women	99.23 ± 9.65	98.27 ± 7.80	99.79 ± 8.56	0.28
Men	102.74 ± 9.03	103.37 ± 8.45	103.24 ± 8.41	0.87
Smokers, % (*n*)				
Current	10.50 (19)	13.55 (34)	10.28 (37)	0.24
Former	26.52 (48)	27.09 (68)	21.67 (78)	
Never smoker	62.98 (114)	59.36 (149)	68.06 (245)	
Physical activity, MET/min/day	264 ± 217	291 ± 311	250 ± 259	0.19
Education level, % (*n*)				
Low-medium	92.82 (168)	94.82 (238)	93.61 (337)	0.68
High	7.18 (13)	5.18 (13)	6.39 (23)	
Prosthesis use, % (*n*)	59.67 (108)	54.18 (136)	46.94 (169)	0.02
**Medication use, % (*n*)**				
Oral antidiabetics	38.12 (69)	34.26 (86)	32.22 (116)	0.39
Insulin	7.73 (14)	5.98 (15)	6.11 (22)	0.72
Hypocholesterolemic agents	38.12 (69)	45.02 (113)	47.22 (170)	0.13
Antihypertensive agents	72.93 (132)	74.50 (187)	75.28 (271)	0.84
**Biochemical Parameters**				
Glucose, mg/dL	123.57 ± 46.66	121.83 ± 36.98	118.47 ± 36.00	0.31
Triglycerides, mg/dL—median (IQR) ^b^	107.60 [76.00–141.97]	120.90 [87.94–154.89]	122.32 [92.73–166.10]	<0.01
Total cholesterol, mg/dL	207.95 ± 39.15	208.38 ± 40.26	209.67 ± 36.62	0.86
HDL-cholesterol, mg/dL—median (IQR)				
Women	58.13 [48.00–65.70]	56.09 [49.90–66.61]	55.34 [48.98–65.57]	0.50
Men	51.85 [44.08–58.80]	48.10 [42.30–55.34]	48.74 [42.00–55.98]	0.03
Systolic blood pressure, mmHg	151.59 ± 19.23	150.46 ± 17.86	149.72 ± 18.26	0.53
Diastolic blood pressure, mmHg	81.83 ± 10.75	82.99 ± 9.24	84.13 ± 9.09	0.03

Data are expressed as mean ± SD for continuous variables and percentages, number (*n*) for categorical variables and median (IQR). ^a^ *p* value was calculated by the chi-square or ANOVA test for categorical and continuous variables, respectively. ^b^ Variables transformed logarithmically before analysis. Abbreviations: MET, Metabolic Equivalent Task; HDL, High Density Lipoprotein; IQR: Interquartile Range

**Table 2:** Dietary characteristics of the study population across self-reported eating speed categories.

	Eating Speed Categories			
	Slow *n* = 181	Medium *n* = 251	Fast *n* = 360	*p*^a^ Value
MedDiet ^b^ adherence (0–14 points)	8.18 ± 1.97	8.34 ± 1.99	8.22 ± 1.83	0.64
Energy intake (Kcal)	2290 ± 512	2271 ± 557	2282 ± 533	0.94
Eating frequency, % (*n*)				
1–2 meals/day	15.17 (27)	12.75 (32)	19.17 (69)	0.10
>3 meals/day	84.83 (151)	87.25 (219)	80.83 (291)	
Macronutrient distribution				
Carbohydrate (g/day)	228 ± 66	227 ± 68	231 ± 72	0.75
Carbohydrate, % of total energy	39.85 ± 6.68	39.84 ± 6.12	40.23 ± 6.57	0.70
Protein (g/day)	93 ± 22	93 ± 21	95 ± 21	0.23
Protein, % of total energy	16.40 ± 2.57	16.59 ± 2.40	17.00 ± 2.58	0.02
Lipid (g/day)	103 ± 26	104 ± 29	103 ± 27	0.95
Lipid, % of total energy	40.77 ± 6.19	41.00 ± 5.93	40.71 ± 6.21	0.84
Dietary fiber (g/day)	23.03 ± 7.54	23.18 ± 7.87	23.68 ± 7.65	0.58
Alcohol intake (g/day)	10.65 ± 17.53	8.88 ± 12.41	7.33 ± 12.36	0.03

Data are expressed as mean ± SD for continuous variables and percentages and numbers (*n*) for categorical variables. ^a^ *p* value was calculated by chi-square or ANOVA test for categorical and continuous variables, respectively. ^b^ Mediterranean Diet.

**Table 3:** Hazard ratio (95% CI) of different cardiovascular risk factors for self-reported eating speed categories.

	Eating Speed Categories		
	Slow	Medium	Fast
	*n* = 181	*n* = 251	*n* = 360
Obesity % (*n*)	41.4 (75)	41 (103)	50.6 (182)
Crude model	1 (Ref.)	0.99 (0.79–1.24)	1.22 (1.00–1.49)
Adjusted model ^a^	1 (Ref.)	0.99 (0.80–1.24)	1.15 (0.94–1.40)
Metabolic syndrome % (*n*)	59.70 (108)	61.00 (153)	64.40 (232)
Crude model	1 (Ref.)	1.02 (0.88–1.19)	1.08 (0.94–1.24)
Adjusted model ^b^	1 (Ref.)	1.02 (0.88–1.18)	0.99 (0.86–1.14)
**Metabolic syndrome components**			
Central obesity % (*n*)	74.60 (135)	74.10 (186)	78.90 (284)
Crude model	1 (Ref.)	0.99 (0.89–1.11)	1.06 (0.95–1.17)
Adjusted model ^b^	1 (Ref.)	1.00 (0.91–1.10)	0.95 (0.87–1.04)
Hypertriglyceridemia % (*n*)	21.60 (39)	29.10 (73)	34.20 (123)
Crude model	1 (Ref.)	1.35 (0.97–1.89)	1.59 (1.16–2.17)
Adjusted model ^b^	1 (Ref.)	1.32 (0.95–1.85)	1.47 (1.08–2.02)
Low HDL-C % (*n*)	22.10 (40)	21.10 (53)	23.90 (86)
Crude model	1 (Ref.)	0.96 (0.67–1.37)	1.08 (0.78–1.50)
Adjusted model ^b^	1 (Ref.)	0.96 (0.68–1.37)	0.94 (0.68–1.30)
High Blood Pressure % (*n*)	96.10 (174)	95.60 (240)	96.40 (347)
Crude model	1 (Ref.)	0.99 (0.96–1.03)	1.00 (0.97–1.04)
Adjusted model ^b^	1 (Ref.)	1.00 (0.96–1.03)	1.00 (0.97–1.04)
High fasting glucose % (*n*)	65.20 (118)	65.70 (165)	61.10 (220)
Crude model	1 (Ref.)	1.01 (0.88–1.16)	0.94 (0.82–1.07)
Adjusted model ^b^	1 (Ref.)	1.00 (0.87–1.15)	0.92 (0.80–1.06)

Abbreviations: CI, confidence interval. ^a^ Model 1 adjusted for sex (female or male), age (years), educational level (low-medium, or high), smoking (current, former, or never), total energy intake, physical activity, oral prosthesis use (yes or no), alcohol consumption (g/d), MedDiet adherence (low or high). ^b^ Model 2 adjusted for variables from model 1. Additionally adjusted by BMI. All analyses were conducted with robust estimates of the variance to correct for intra-cluster correlation in the Cox regression models.

## Supplementary Materials

The following are available online at http://www.mdpi.com/2072-6643/11/1/83/s1, Table S1: Hazard ratio (95% IC) of different cardiovascular risk factors for self-reported eating speed categories.

## References

[1] Grundy S.M., Cleeman J.I., Daniels S.R., Donato K.A., Eckel R.H., Franklin B.A., Gordon D.J., Krauss R.M., Savage P.J., Smith S.C. (2005). Diagnosis and management of the metabolic syndrome: An American Heart Association/National Heart, Lung, and Blood Institute Scientific Statement. Circulation.

[2] Krauss R.M., Eckel R.H., Howard B., Appel L.J., Daniels S.R., Deckelbaum R.J., Erdman J.W., Kris-Etherton P., Goldberg I.J., Kotchen T.A. (2000). AHA Dietary Guidelines: Revision 2000: A statement for healthcare professionals from the Nutrition Committee of the American Heart Association. Stroke.

[3] Robinson E., Almiron-Roig E., Rutters F., de Graaf C., Forde C.G., Tudur Smith C., Nolan S.J., Jebb S.A. (2014). A systematic review and meta-analysis examining the effect of eating rate on energy intake and hunger 1–3. Am. J. Clin. Nutr..

[4] St-Onge M.P., Ard J., Baskin M.L., Chiuve E., Johnson H.M., Kris-Etherton P., Varady K. (2017). Meal Timing and Frequency: Implications for cardiovascular disease prevention: A scientific statement from the American Heart Association. Circulation.

[5] Ritchie L.D. (2012). Less frequent eating predicts greater BMI and waist circumference in female adolescents. Am. J. Clin. Nutr..

[6] Chen H.J., Wang Y., Cheskin L.J. (2017). Relationship between frequency of eating and cardiovascular disease mortality in U.S. adults: The NHANES III follow-up study. Ann. Epidemiol..

[7] Leong S.L., Madden C., Gray A., Waters D., Horwath C. (2011). Faster Self-Reported Speed of Eating Is Related to Higher Body Mass Index in a Nationwide Survey of Middle-Aged Women. J. Am. Diet. Assoc..

[8] Otsuka R., Tamakoshi K., Yatsuya H., Murata C., Sekiya A., Wada K., Zhang H.M., Matsushita K., Sugiura K., Takefuji S. (2006). Eating fast leads to obesity: Findings based on self-administered questionnaires among middle-aged Japanese men and women. J. Epidemiol..

[9] Lee K.S., Kim D.H., Jang J.S., Nam G.E., Shin Y.N., Bok A.R., Kim M.J., Cho K.H. (2013). Eating rate is associated with cardiometabolic risk factors in Korean adults. Nutr. Metab. Cardiovasc. Dis..

[10] van den Boer J.H.W., Kranendonk J., van de Wiel A., Feskens E.J.M., Geelen A., Mars M. (2017). Self-reported eating rate is associated with weight status in a Dutch population: A validation study and a cross-sectional study. Int. J. Behav. Nutr. Phys. Act..

[11] Leong S.L., Gray A., Horwath C.C. (2016). Speed of eating and 3-year BMI change: A nationwide prospective study of mid-age women. Public Health Nutr..

[12] Tanihara S., Imatoh T., Miyazaki M., Babazono A., Momose Y., Baba M., Uryu Y., Une H. (2011). Retrospective longitudinal study on the relationship between 8-year weight change and current eating speed. Appetite.

[13] Hurst Y., Fukuda H. (2018). Effects of changes in eating speed on obesity in patients with diabetes: A secondary analysis of longitudinal health check-up data. BMJ Open.

[14] Ohkuma T., Hirakawa Y., Nakamura U., Kiyohara Y., Kitazono T., Ninomiya T. (2015). Association between eating rate and obesity: A systematic review and meta-analysis. Int. J. Obes..

[15] Karl J.P., Young A.J., Rood J.C., Montain S.J. (2013). Independent and Combined Effects of Eating Rate and Energy Density on Energy Intake, Appetite, and Gut Hormones. Obes. Biol. Integr. Physiol..

[16] Otsuka R., Tamakoshi K., Yatsuya H., Wada K., Matsushita K., OuYang P., Hotta Y., Takefuji S., Mitsuhashi H., Sugiura K. (2008). Eating fast leads to insulin resistance: Findings in middle-aged Japanese men and women. Prev. Med..

[17] Tao L., Yang K., Huang F., Liu X., Li X., Luo Y., Wu L., Guo X. (2018). Association between self-reported eating speed and metabolic syndrome in a Beijing adult population: A cross-sectional study. BMC Public Health.

[18] Estruch R., Ros E., Salas-Salvadó J., Covas M.I., Corella D., Arós F., Gómez-Gracia E., Ruiz-Gutiérrez V., Fiol M., Lapetra J. (2018). Primary Prevention of Cardiovascular Disease with a Mediterranean Diet Supplemented with Extra-Virgin Olive Oil or Nuts. N. Engl. J. Med..

[19] Estruch R., Martínez-González M.A., Corella D., Salas-Salvadó J., Ruiz-Gutiérrez V., Covas M.I., Fiol M., Gómez-Gracia E., López-Sabater M.C., Vinyoles E. (2006). Effects of a Mediterranean-style diet on cardiovascular risk factors: A randomized trial. Ann. Intern. Med..

[20] Martínez-González M.Á., Corella D., Salas-Salvadó J., Ros E., Covas M.I., Fiol M., Wärnberg J., Arós F., Ruíz-Gutiérrez V., Lamuela-Raventós R.M. (2012). Cohort profile: Design and methods of the PREDIMED study. Int. J. Epidemiol..

[21] Martínez-González M.A., García-Arellano A., Toledo E., Salas-Salvadó J., Buil-Cosiales P., Corella D., Covas M.I., Schröder H., Arós F., Gómez-Gracia E. (2012). A 14-Item Mediterranean Diet Assessment Tool and Obesity Indexes among High-Risk Subjects: The PREDIMED trial. PLoS ONE.

[22] Schröder H., Fitó M., Estruch R., Martínez-González M.A., Corella D., Salas-Salvadó J., Lamuela-Raventós R., Ros E., Salaverría I., Fiol M.J. (2011). A Short Screener Is Valid for Assessing Mediterranean Diet Adherence among Older Spanish Men and Women. J. Nutr..

[23] Fernández-Ballart J.D., Piñol J.L., Zazpe I., Corella D., Carrasco P., Toledo E., Perez-Bauer M., Martínez-González M.A., Salas-Salvadó J., Martín-Moreno J.M. (2010). Relative validity of a semi-quantitative food-frequency questionnaire in an elderly Mediterranean population of Spain. Br. J. Nutr..

[24] Elosua R., Marrugat J., Molina L., Pons S., Pujol E. (1994). Validation of the Minnesota Leisure Time Physical Activity Questionnaire in Spanish men. The MARATHOM Investigators. Am. J. Epidemiol..

[25] Elosua R., Garcia M., Aguilar A., Molina L., Covas M.I., Marrugat J. (2000). Validation of the Minnesota Leisure Time Physical Activity Questionnaire In Spanish Women. Investigators of the MARATDON Group. Med. Sci. Sports Exerc..

[26] Salas-Salvadó J., Rubio M.A., Barbany M., Moreno B., Grupo Colaborativo de la SEEDO (2007). SEEDO 2007 Consensus for the evaluation of overweight and obesity and the establishment of therapeutic intervention criteria. Med. Clin..

[27] Alberti K.G., Eckel R.H., Grundy S.M., Zimmet P.Z., Cleeman J.I., Donato K.A., Fruchart J.C., James W.P.T., Loria C.M., Smith S.C. (2009). Harmonizing the metabolic syndrome: A joint interim statement of the international diabetes federation task force on epidemiology and prevention; National heart, lung, and blood institute; American heart association; World heart federation; International atherosclerosis society; and international association for the study of obesity. Circulation.

[28] Barros A.J., Hirakata V.N. (2003). Alternatives for logistic regression in cross-sectional studies: An empirical comparison of models that directly estimate the prevalence ratio. BMC Med. Res. Methodol..

[29] Tanaka Y., Wake N., Kato K. (2009). Letters to the Editor. Menopause.

[30] Maruyama K., Sato S., Ohira T., Maeda K., Noda H., Kubota Y., Nishimura S., Kitamura A., Kiyama M., Okada T. (2008). The joint impact on being overweight of self reported behaviours of eating quickly and eating until full: Cross sectional survey. BMJ.

[31] Lee J.S., Mishra G., Hayashi K., Watanabe E., Mori K., Kawakubo K. (2016). Combined eating behaviors and overweight: Eating quickly, late evening meals, and skipping breakfast. Eat. Behav..

[32] Saito A., Kawai K., Yanagisawa M., Yokoyama H., Kuribayashi N., Sugimoto H., Oishi M., Wada T., Iwasaki K., Kanatsuka A. (2012). Self-reported rate of eating is significantly associated with body mass index in Japanese patients with type 2 diabetes. Japan Diabetes Clinical Data Management Study Group (JDDM26). Appetite.

[33] Kral J.G., Buckley M.C., Kissileff H.R., Schaffner F. (2001). Metabolic correlates of eating behavior in severe obesity. Int. J. Obes..

[34] Zhu B., Haruyama Y., Muto T., Yamazaki T. (2015). Association between eating speed and metabolic syndrome in a three-year population-based cohort study. J. Epidemiol..

[35] Yamane M., Ekuni D., Mizutani S., Kataoka K., Sakumoto-Kataoka M., Kawabata Y., Omori C., Azuma T., Tomofuji T., Iwasaki Y. (2014). Relationships between eating quickly and weight gain in Japanese University students: A longitudinal study. Obesity.

[36] Toyama K., Zhao X., Kuranuki S., Oguri Y., Kashiwa E., Yoshitake Y., Nakamura T. (2015). The effect of fast eating on the thermic effect of food in young Japanese women. Int. J. Food Sci. Nutr..

[37] Shah M., Crisp K., Adams-Huet B., Dart L., Bouza B., Franklin B., Phillips M. (2015). The Effect of Eating Speed at Breakfast on Appetite Hormone Responses and Daily Food Consumption. J. Investig. Med..

[38] Shah M., Copeland J., Dart L., Adams-Huet B., James A., Rhea D. (2014). Slower eating speed lowers energy intake in normal-weight but not overweight/obese subjects. J. Acad. Nutr. Diet..

[39] Lee S., Ko B.J., Gong Y., Han K., Lee A., Han B.D., Yoon Y.J., Park S., Kim J.H., Mantzoros C.S. (2016). Self-reported eating speed in relation to non-alcoholic fatty liver disease in adults. Eur. J. Nutr..

