# Supplementary material for: Association between Eating Speed and Classical Cardiovascular Risk Factors: A Cross-Sectional Study
Source: Nutrients. 2019 Jan 4;11(1):83. doi: 10.3390/nu11010083 (PMC6356451; doi:10.3390/nu11010083)
Supplement: Supplementary file 1 [file nutrients-11-00083-s001.pdf]

Supplementary file

Table S1. Hazard ratio (95% IC) of different cardiovascular risk factors for self-reported eating speed categories

	**Eating Speed Categories**		
	**Slow**	**Medium**	**Fast**
	***n* = 181**	***n* = 251**	***n* = 360**
Obesity % (*n*)	41.4 (75)	41 (103)	50.6 (182)
Crude model	1 (Ref.)	0.99 (0.79–1.24)	1.22 (1.00–1.49)
Adjusted model [a]	1 (Ref.)	1.00 (0.80–1.24)	1.15 (0.94–1.41)
Metabolic syndrome % (*n*)	59.70 (108)	61.00 (153)	64.40 (232)
Crude model	1 (Ref.)	1.02 (0.88–1.19)	1.08 (0.94–1.24)
Adjusted model [b]	1 (Ref.)	1.02 (0.88–1.18)	0.99 (0.86–1.14)
**Metabolic syndrome components**			
Central obesity % (*n*)	74.60 (135)	74.10 (186)	78.90 (284)
Crude model	1 (Ref.)	0.99 (0.89–1.11)	1.06 (0.95–1.17)
Adjusted model [c]	1 (Ref.)	1.00 (0.91–1.10)	0.95 (0.87–1.04)
Hypertriglyceridemia % (*n*)	21.60 (39)	29.10 (73)	34.20 (123)
Crude model	1 (Ref.)	1.35 (0.97–1.89)	1.59 (1.16–2.17)
Adjusted model [c]	1 (Ref.)	1.36 (0.99–1.86)	1.55 (1.15–2.08)
Low HDL-C % (*n*)	22.10 (40)	21.10 (53)	23.90 (86)
Crude model*	1 (Ref.)	0.96 (0.67–1.37)	1.08 (0.78–1.50)
Adjusted model [c]	1 (Ref.)	0.92 (0.66–1.28)	0.88 (0.65–1.18)
High Blood Pressure % (*n*)	96.10 (174)	95.6 0(240)	96.40 (347)
Crude model	1 (Ref.)	0.99 (0.96–1.03)	1.00 (0.97–1.04)
Adjusted model [c]	1 (Ref.)	0.99 (0.96–1.03)	1.00 (0.97–1.03)
High fasting glucose % (*n*)	65.20 (118)	65.70 (165)	61.10 (220)
Crude model	1 (Ref.)	1.01 (0.88–1.16)	0.94 (0.82–1.07)
Adjusted model [c]	1 (Ref.)	1.00 (0.87–1.15)	0.93 (0.81–1.07)

Abbreviations: CI, confidence interval.

Cox regression models were applied with robust estimates of the variance to correct for intra-cluster correlation

[a] Model adjusted for sex (female or male), age (years), educational level (low-medium and high), smoking (current, former, or never), total energy intake, physical activity, oral prosthesis use (yes or no), alcohol consumption (g/d), MedDiet Adherence (low or high), high blood pressure (yes or no), and high fasting glucose (yes or no).

[b] Model 2 adjusted for variables from model 1 except for high blood pressure (yes or no) and high fasting glucose (yes or no). Additionally adjusted by BMI.

[c] Model 3 adjusted for variables from model 2 plus the other four metabolic syndrome individual components.
